# FOXQ1 inhibits the progression of osteoarthritis by regulating pyroptosis

**DOI:** 10.18632/aging.205600

**Published:** 2024-03-19

**Authors:** Zhihuan Luo, Hui Zeng, Kanghua Yang, Yihai Wang

**Affiliations:** 1 Department of Sports Medicine, Ganzhou People’s Hospital, Ganzhou 341000, Jiangxi Province, China

**Keywords:** FOXQ1, osteoarthritis, inflammasome, pyroptosis

## Abstract

Background: Osteoarthritis (OA) is the most common age-related joint disease, and the NLRP3-induced pyroptosis has been demonstrated in its progression. The upstream molecules or specific mechanisms controlling NLRP3 and pyroptosis in OA remain unclear.

Methods: Transcriptome sequencing was performed in the OA mice model, and the expression levels of differentially expressed genes were assessed by qRT-PCR. The cell model was constructed by IL-1β-induced ATDC5 cells. The cell proliferation was examined using CCK-8 assay, and apoptosis was tested using flow cytometry. Western blot was used in protein inspection, and ELISA was used in inflammatory response evaluation.

Results: Compared with the control group, there were 229 up-regulated and 32 down-regulated genes in model group. We detected that FOXQ1 was down-regulated in the OA mice model, improved proliferation, and restrained apoptosis of chondrocytes. Over-expression of FOXQ1 could inhibit pyroptosis-related proteins and inflammatory cytokines, containing NLRP3, Caspase-1, GSDMD, IL-6, IL-18, and TNF-α, and in contrast, FOXQ1 silencing exerted the opposite trend.

Conclusions: FOXQ1 may inhibit OA progression via down-regulating NLRP3-induced pyroptosis in the present study.

## INTRODUCTION

Osteoarthritis (OA) is the most familiar joint disease and influences 250 million people worldwide [1]. Due to the aging of population, approximately 7% of the global population suffers from OA [2]. The pathological modifications related to OA play a role in all tissues in the joint and encompass cartilage degeneration, subchondral sclerosis, variable levels of synovial inflammation, osteophyte formation, and hypertrophy of the total joint pill [3]. There are many risk factors that can affect OA, containing age, gender, obesity, diabetes, weight-bearing work, hereditary, exercise, cardiovascular disease, hip deformities, depression, hypertension and so on [1]. Meanwhile, with the development of mechanism studies, there is increasing concern about the impact of inflammasomes in OA [4].

Inflammasomes, which are stimulated by nuclear factor kappa B (NF-κB) signaling, can transform interleukin-1β (IL-1β) and interleukin-18 (IL-18) to mature proinflammatory cytokines. Thus, inflammasomes are deemed as the causes of the low-grade inflammatory pathology occurrence [5]. NLR family pyrin domain containing 3 (NLRP3), apoptosis-related speck-like protein containing (ASC) a caspase recruitment domain (CARD) and pro-caspase-1 are members of inflammasomes [6]. It has been demonstrated that Ox-LDL [7], LPS [8], and other specific pathogens or danger-associated molecular patterns can activate chondrocyte pyroptosis, a sort of programmed cell death executed by gasdermin family members [9], via the NLRP3 pathway [10, 11]. The pyroptosis in articular cartilage contributes to the annihilation of chondrocytes, dissolve of the intrinsic structurally steady cartilage surrounding the extracellular matrix, and release of proinflammatory factors, which indirectly expedites cartilage degeneration and facilitates joint inflammation [12].

Some pyroptosis-regulated genes have been identified that lead to the progression of OA, including TLR4 [13], P2X7 [14], TNF [15], and SDF1 [16]. The upstream molecules or specific mechanisms controlling NLRP3 and pyroptosis in OA remain unclear. Improving the understanding of inflammation and inflammation-induced pyroptosis in OA may help to find novel therapeutic targets. In the present study, we evaluated the differences between the OA mice model and the control group by transcriptome analysis, and identified transcription factor forkhead box Q1 (FOXQ1) as a novel OA inhibitor. We also investigated the mechanism of FOXQ1 inhibiting the progression of OA by regulating pyroptosis through cell experiments.

## MATERIALS AND METHODS

### Construction of mouse OA model

Balb/c mice (SPF Biotechnology Co., Ltd. Beijing, China) had been grouped into control and model randomly. The DMM (destabilization of the medial meniscus) model in mice is used as surgical model for OA. All of them were kept in a 12 h light/dark cycle with unlimited access to standard mouse food and water. They were divided into a control group (sham) and a model group (OA) randomly, with 6 mice in every group. DMM or sham surgical operation was once carried out on mice aged 12 weeks. The medial meniscotibial ligament (MMTL) is transected to generate DMM on the left knee joint [17]. After the surgery to prevent joint infection, the subcutaneous injection of Amoxicillin (20 mg/kg) (Novopharm, Toronto, Ontario, Canada) was carried out. Buprenorphine (0.05 mg/kg) (Schering-Plough, Hertfordshire, UK) was subcutaneously injected at 0 and 4 h after surgery. To encourage exercise, a running wheel was mounted on the third day later when the surgery was finished. The mice were euthanized through inhalation of isoflurane for 2-3 min. The blood and knee joints tissue samples were obtained from two groups.

### Histological analysis

Knee joints were extracted and fixed in 4% paraformaldehyde for 24 h. Then they were put in 10% EDTA with 0.1 M phosphate buffer (pH = 7.4) to decalcify for 3 weeks. Tissues were fixed in paraffin and cut into 6 μm-thick sections, which were stained subsequently with hematoxylin-eosin (HE). Image Pro-Plus 6.0 software (Media Cybernetics, Rockville, MD, USA) was used in measuring the thickness of hyaline cartilage and calcified cartilage, according to the tidemark position, based on HE staining results.

### TUNEL staining

TUNEL apoptosis assay kit (Beyotime, Shanghai, China) was used to assess the apoptosis of articular chondrocytes. Paraffin sections were dewaxed in xylene and washed three times with PBS. The sections were incubated with proteinase K at 37° C for 30 min. Clean again with PBS. Then the sections were incubated with H2O2 at room temperature for 10 min. After that, the TUNEL reagents were added and incubated at 37° C in dark for 60 min. Finally, DAB was added for color development. The sections were stained with hematoxylin and then sealed. Fluorescence microscope (Olympus, Japan) was used in apoptotic articular chondrocytes counting.

### RNA isolation, transcriptome sequencing, and data analysis

Knee joints RNA was extracted with TRIzol™ Reagent (Invitrogen, USA). We obtained the sequencing libraries from 2 μg of RNA per sample with the NEBNext® UltraTM RNA Library Prep Kit for Illumina® (NEB, USA). Transcriptome sequencing was performed on Illumina Novaseq platform, and 150-bp paired-end reads were produced. Paired-end clean reads were aligned to mm10 using HISAT2 v 2.0.5.

The reads of each gene were counted by HTseq v 2.0.2, and the fragments per kilobase of transcript sequence per million base pairs sequenced (FPKM) was used in analysis of expression with the R package limma [18]. Differentially expressed genes (DEGs) were confirmed with *p*-value ≤ 0.05 and |log2 Fold Change| (|log2FC|) ≥ 1.

The R package Pheatmap was used in bidirectional clustering analysis on the union and samples of all two groups of DEGs. Clustering was carried out according to the expression of the same gene in different samples and the expression pattern of DEGs in the same sample. Complete linkage, based on Euclidean distance, was used to perform the clustering.

### qPCR

The Roche Light-Cycler 480 Real-Time PCR system was utilized to evaluate the RNA transcription level of the ATDC5 cell model and articular cartilage tissue of the OA mice model or controls. SYBRTM Green PCR Master Mix (4309155, Thermo Fisher, Waltham, MA, USA) was used for qPCR (total reaction volume, 20 μL). The program was shown as follows: 10 min, 95° C, (15 s, 95° C; 30 s, 72° C), 40 cycles. Quantification was performed with the 2-^ΔΔCT^ approach. GAPDH was chosen as the internal control. The sequences of primers used were listed in Supplementary Table 1.

### Cell culture

The ATDC5 cells were cultured in Dulbecco’s modified eagle medium (DMEM) (Thermo Fisher) with 10% fetal bovine serum (FBS) (Thermo Fisher). The control was treated with phosphate buffer saline (PBS) (Thermo Fisher) for 24 h, and the model group was stimulated with IL-1β (SRP3083, Sigma, USA) in doses with 0, 2.5, 5 and 10 ng/mL for 24 h. The overexpression vector of FOXQ1 (oe-FOXQ1) and corresponding negative control (oe-NC) were transfected into ATDC5 cells with Lipofectamine™ 3000 (Thermo Fisher), respectively. Additionally, FOXQ1 knockdown in ATDC5 cells was achieved by transfection with FOXQ1 siRNAs (si-FOXQ1) using Lipofectamine™ 3000 (Thermo Fisher). The overexpression vectors were acquired from Hanheng Biotechnology Co., Ltd. (Shanghai, China), and the siRNA sequences were designed by GenePharma Co., Ltd (Shanghai, China). The sequences of FOXQ1 siRNAs were provided in Supplementary Table 1.

### Enzyme-linked immunosorbent assay (ELISA)

ATDC5 cells (5 × 10^4^) were seed into 24-well plates and cultured for 72 h when the transfection or treatment finished. Then, we collected the medium and applied them to evaluate the levels of IL-1β, IL-18, IL-6 and tumor necrosis factor α (TNF-α) with the specific ELISA kit (Esebio, Shanghai, China). The inflammatory factors of mouse serum were also detected by ELISA assay. The concentration of these cytokines was detected by Multiskan FC (Thermo Fisher).

### Cell proliferation assay

ATDC5 cells (4 × 10^3^) were seeded in 96-well plates. The cell viability was detected using a Cell Counting Kit-8 (CCK-8) assay (Beyotime). CCK-8 assay was performed to detect the proliferation of ATDC5 cells stimulated by IL-1β at different concentrations (0, 2.5, 5, 10 ng/mL) after 24 h. Moreover, after transfection, the proliferation of ATDC5 cells in each group was detected. After adding 10 μL CCK-8 to each well, cells were incubated at 37° C for 2 h and OD_450_ was measured by Multiskan FC (Thermo Fisher).

### Flow cytometry

Cell apoptosis was tested with Annexin V-FITC apoptosis kit (V13242, Thermo Fisher). ATDC5 cells (2 × 10^5^) were seeded into 6-well plates and incubated for 72 h when they were the indicated transfection or treatment. Cells were collected and interacted with Annexin V-FITC binding buffer, followed by dying with Annexin V-FITC and propidium iodide. The apoptotic cells were detected with NovoCyte 1050 (Agilent, Hangzhou, China). The apoptotic rate represented the proportion of apoptotic cells.

### Western blot

RIPA Lysis buffer (R0010, Solarbio, Beijing, China) was used to extract the cells’ protein, BCA Protein Assay Kit (P0012S, Beyotime) was employed to detect quantities.

Cells’ protein was separated via SDS-PAGE electrophoresis (P0508S, Beyotime), and transferred to PVDF membranes (1620177, Bio-Rad, Hercules, CA, USA). The membrane was blocked by 5% fat-free milk, then interacted with primary antibodies for NLRP3 (ab263899, 1:1000, Abcam, Cambridge, MA, USA), Caspase-1 (ab207802, 1:1000, Abcam), GSDMD (ab219800, 1:1000, Abcam) overnight at 4° C, and IgG conjugated via HRP (ab6721, 1:10000, Abcam) for 2 h at room temperature. The blots were exposed to ECL reagent (Abcam) and the images were obtained using Quantity One (Bio-Rad, Hercules, CA, USA).

### Statistical analysis

The results were presented as the means ± SDs. Differences between two groups were evaluated using a two-tailed Student’s t-test, and the One-way ANOVA was used for assessing comparisons among groups followed by Tukey’s test. *P* < 0.05 was considered statistically significant.

### Availability of data and material

The datasets generated and/or analysed during the current study are available in the [NCBI] repository, [http://www.ncbi.nlm.nih.gov/bioproject/951274].

## RESULTS

### Construction of OA mouse model

**Figure 1:**
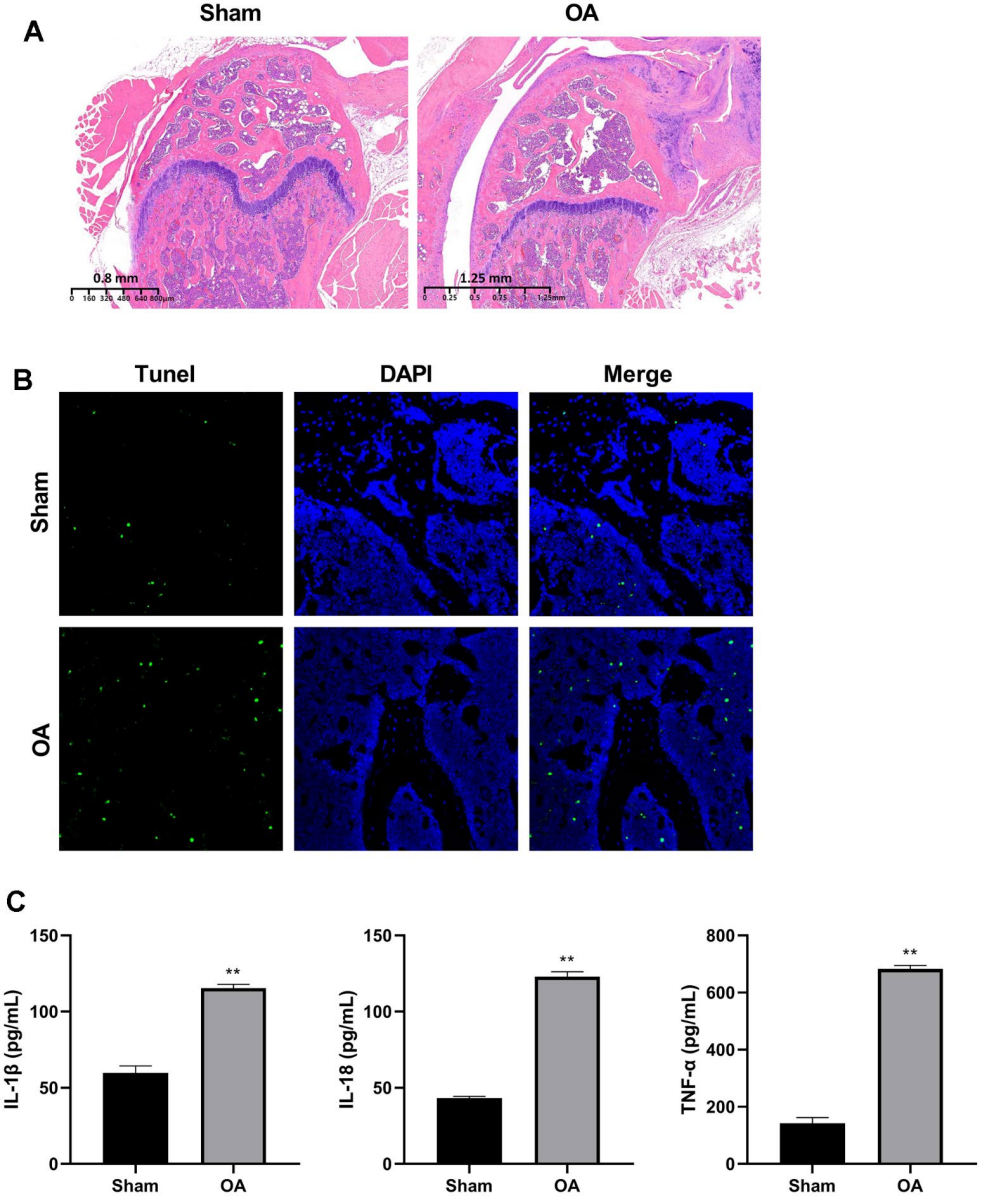
**Construction of osteoarthritis mice model.** (**A**) Cartilage HE staining results of OA model and controls. (**B**) Apoptosis of chondrocytes was detected by TUNEL immunofluorescence. (**C**) ELISA was used to detect the expression of IL-1β, IL-18, and TNF-α in the serum of mice models and controls. (^**^P < 0.01 vs. sham, n = 6).

To detect the RNA transcriptome difference between OA cartilage tissue and controls, we constructed the DMM model as an OA mice model. HE staining showed that the articular cartilage of OA group was thinner, the layer was clear, and the chondrocytes were irregularly arranged in comparison with the sham group (Figure 1A). The TUNEL results showed obvious chondrocytes apoptosis in the OA group (Figure 1B).

The serum of IL-1β, IL-18, and TNF-α levels were significantly elevated in the OA group (*P* < 0.01) (Figure 1C).

### Data analysis of transcriptome sequencing

**Figure 2:**
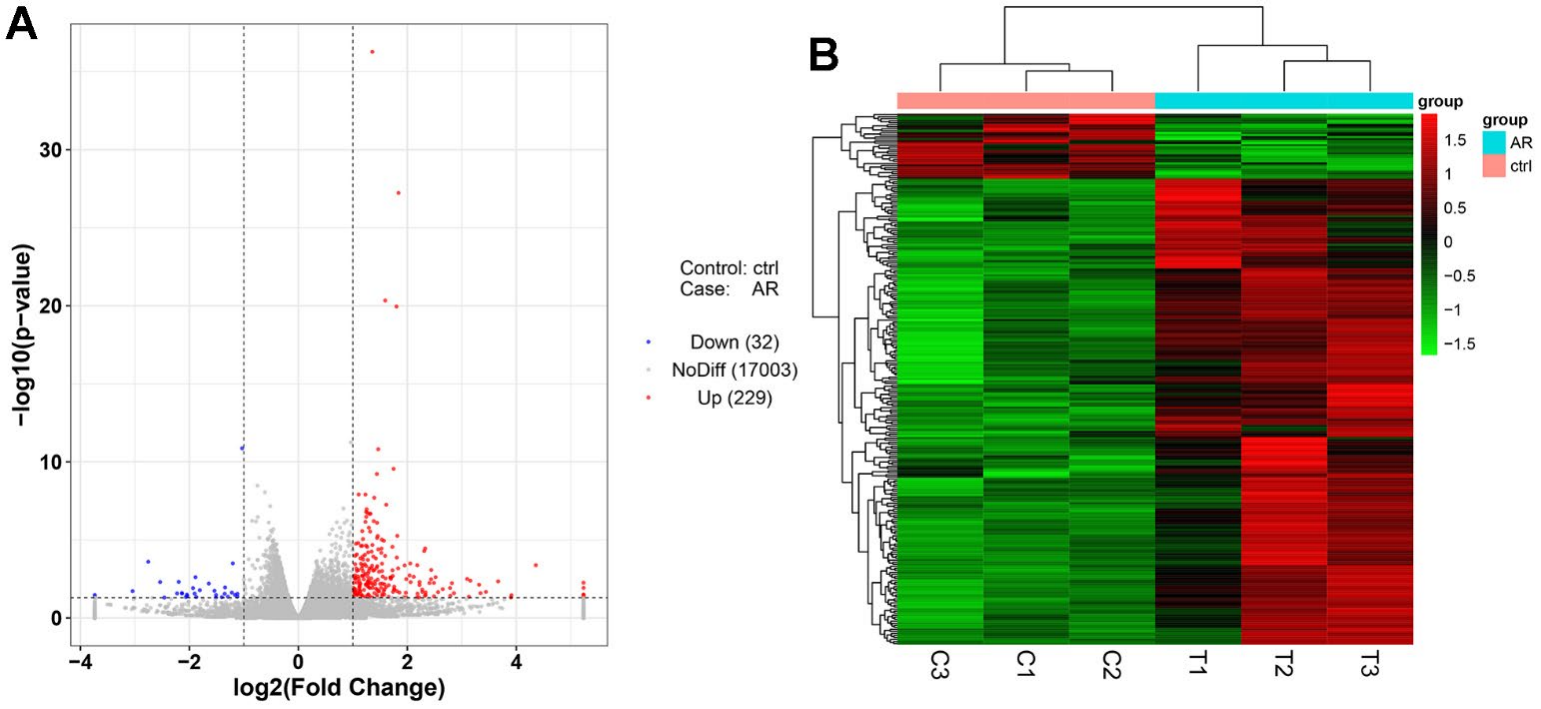
**Differentially expressed gene analysis of the control group and model group.** (**A**) Volcano map of DEGs. (**B**) Heatmap of DEGs.

**Figure 3:**
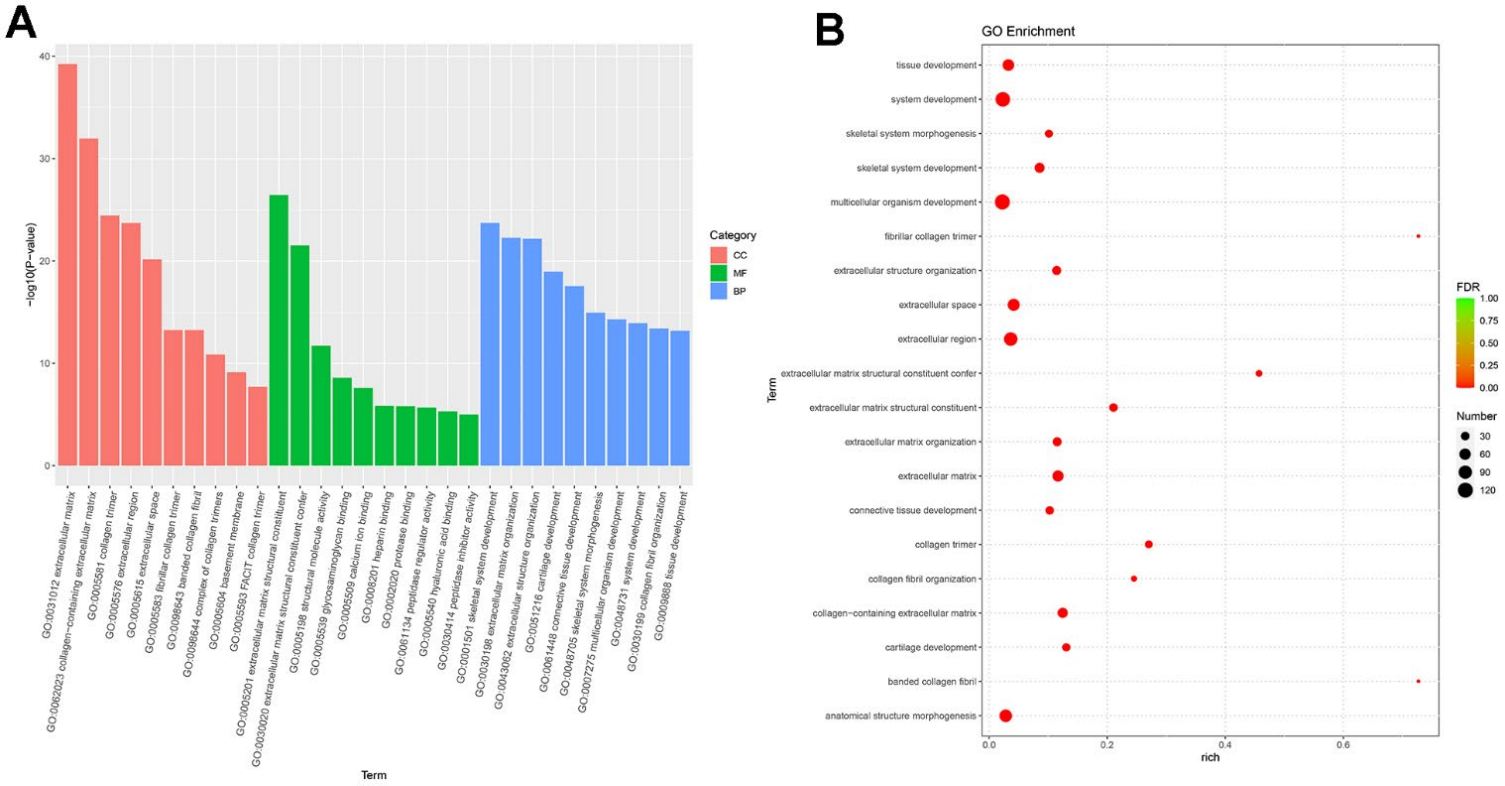
**GO enrichment analysis of differential expressed genes.** (**A**) Bar plot of GO enrichment. (**B**) Bubble plot of GO enrichment.

**Figure 4:**
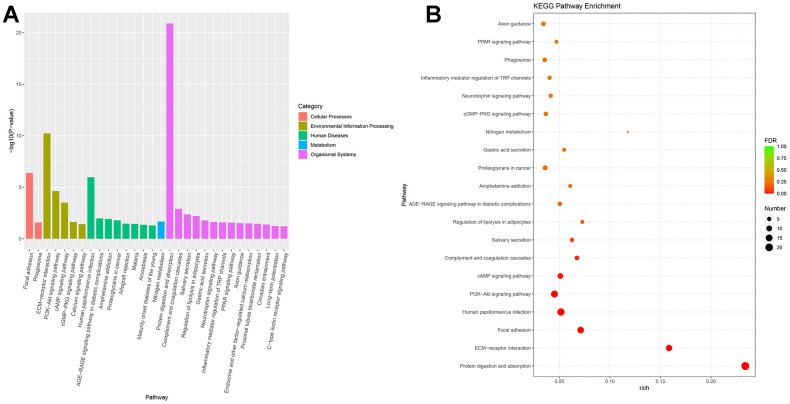
**KEGG enrichment of differential expressed genes.** (**A**) Bar plot of KEGG enrichment. (**B**) Bubble plot of KEGG enrichment.

Next-generation sequencing (NGS) was used in RNA transcription level difference detection in the OA model and the control group. We detected that a total of 229 genes were up-regulated and 32 genes were down-regulated in the model group relative to the control group (*p*-value ≤ 0.05 and |log2FC| ≥ 1) (Figure 2A, 2B). The top 15 up- and down-regulated genes were respectively shown in Supplementary Table 2. GO enrichment analysis results showed that DEGs were enriched in system development, multicellular organism development and extracellular region, anatomical structure morphogenesis, and so on (Figure 3A, 3B). KEGG enrichment analysis detected that DEGs focused on protein digestion and absorption, ECM-receptor interaction, adhesion plaque, HPV infection, PI3K-AKT signaling pathway, and cAMP signaling pathway (Figure 4A, 4B).

### Validation of DEGs

**Figure 5:**
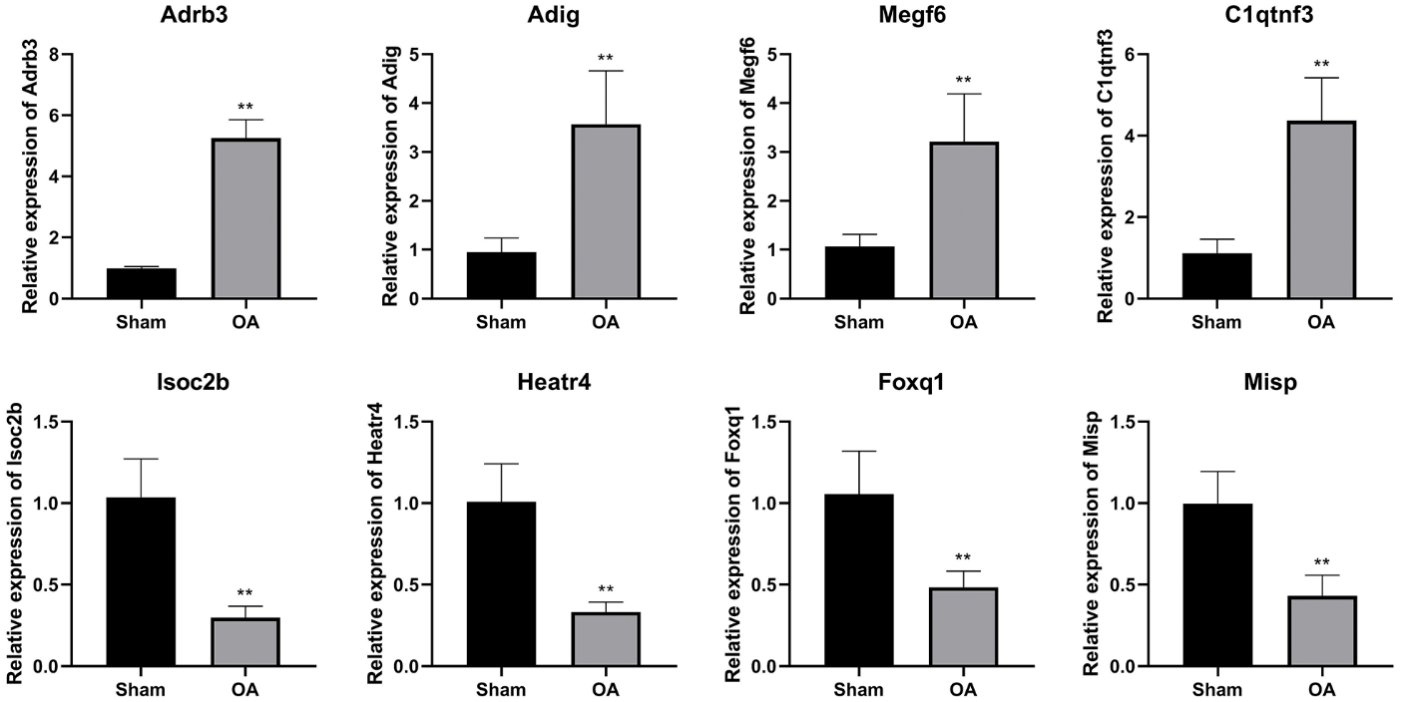
**QRT-PCR verification of differential expressed genes.** (^**^P < 0.01 vs. sham, n = 6).

To confirm the NGS result, eight candidate DEGs were validated by qRT-PCR. The expressions of *Adrb3*, *Adig*, *Megf6,* and *C1qtnf3* were increased, while the levels of *Isoc2b*, *Heatr4*, *Foxq1,* and *Misp* were inhibited in the OA group (*P* < 0.01, Figure 5).

OA is a typical age-related immune disease, and FOXQ1 is involved in the regulation of senescence-associated inflammation [19]. Moreover, FOXQ1 has been a potential prognostic and biomarker for a variety of cancers, such as colorectal cancer and breast cancer [20–22], but the research on orthopedic disease is extremely rare. In recent years, studies have found that FOXQ1 has the potential to promote the osteogenic differentiation of bone mesenchymal stem cells [23–25]. Therefore, FOXQ1 was selected as the target gene for further study to clarify the effect on the progression of OA.

### Effects of FOXQ1 in IL-1β treated ATDC5 cell model

**Figure 6:**
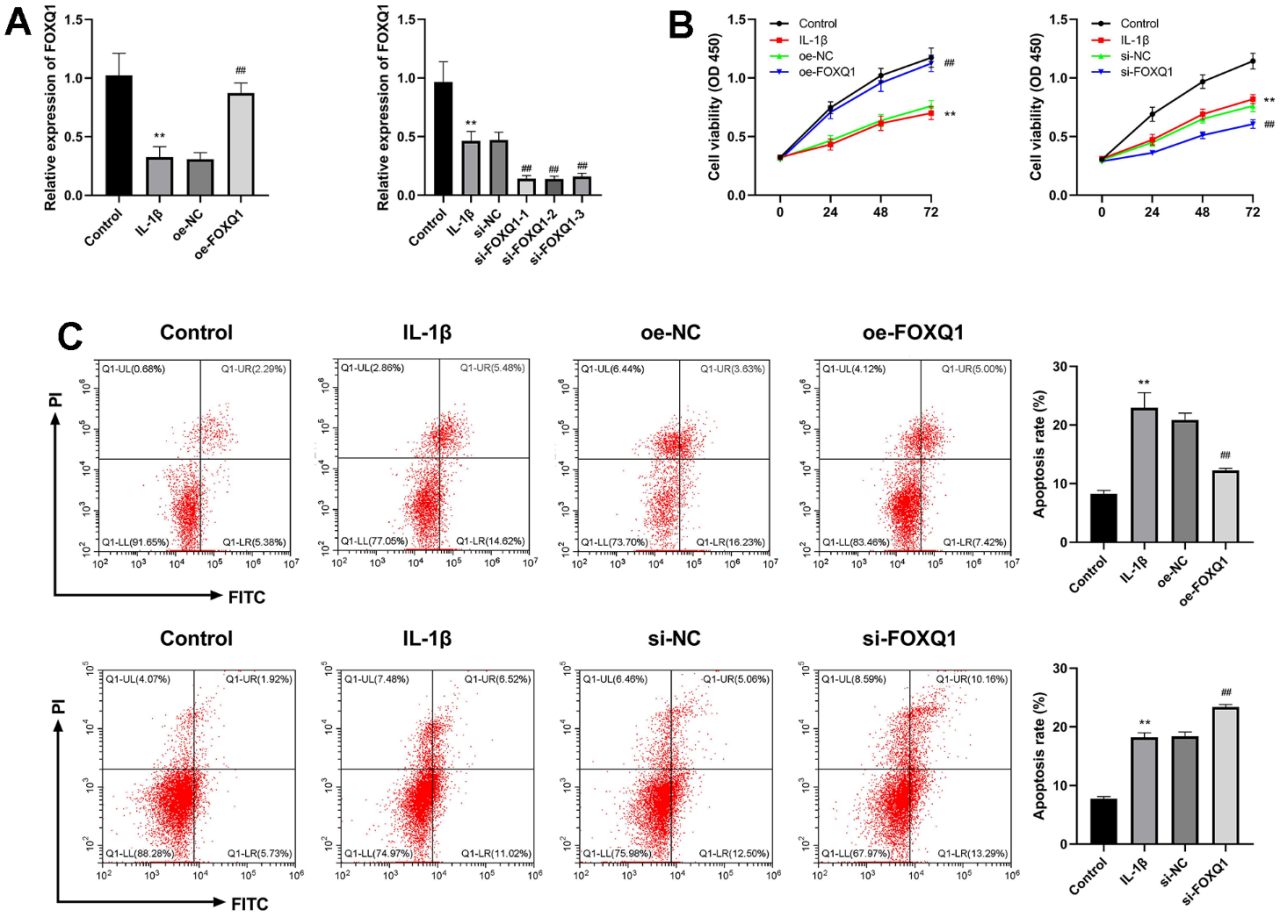
**Effects of FOXQ1 in IL-1β treated ATDC5 cell model.** (**A**) Overexpression of FOXQ1 and FOXQ1 knockdown were determined by qRT-PCR assays. (**B**) CCK-8 results. (**C**) Flow cytometry detecting apoptosis. (^**^P < 0.01 vs. Control; ^##^ *P* < 0.01 vs. IL-1β).

To assess the effect of FOXQ1 on OA, the ATDC5 cells were treated with IL-1β to establish a OA cell model, then were transfected with FOXQ1 overexpression vector, siRNAs and corresponding negative controls. The cells were divided into IL-1β+oe-NC, IL-1β+oe-FOXQ1, IL-1β+si-NC, IL-1β+si-FOXQ1 group. The results of qRT-PCR assay demonstrated that FOXQ1 expression was decreased in the IL-1β group (*P* < 0.01), and obviously upregulated in IL-1β+oe-FOXQ1 group, indicating FOXQ1 overexpression cell model was successfully (*P* < 0.01). Besides, FOXQ1 expression was significantly downregulated when transfected with si-FOXQ1-1, so si-FOXQ1-1 was selected for further experiments (*P* < 0.01, Figure 6A). The cell viability of the IL-1β group was decreased compared with the control group (*P* < 0.01). FOXQ1 overexpression enhanced, and FOXQ1 silencing inhibited the cell viability of ATDC5 cells treated with IL-1β (*P* < 0.01, Figure 6B). In addition, the results of apoptosis showed that the apoptosis rate of the IL-1β group was higher than that of the control group (*P* < 0.01). FOXQ1 overexpression decreased the number of apoptotic cells, and downregulation of FOXQ1 led to an increase in apoptotic cells (*P* < 0.01, Figure 6C).

### FOXQ1 inhibits pyroptosis

**Figure 7:**
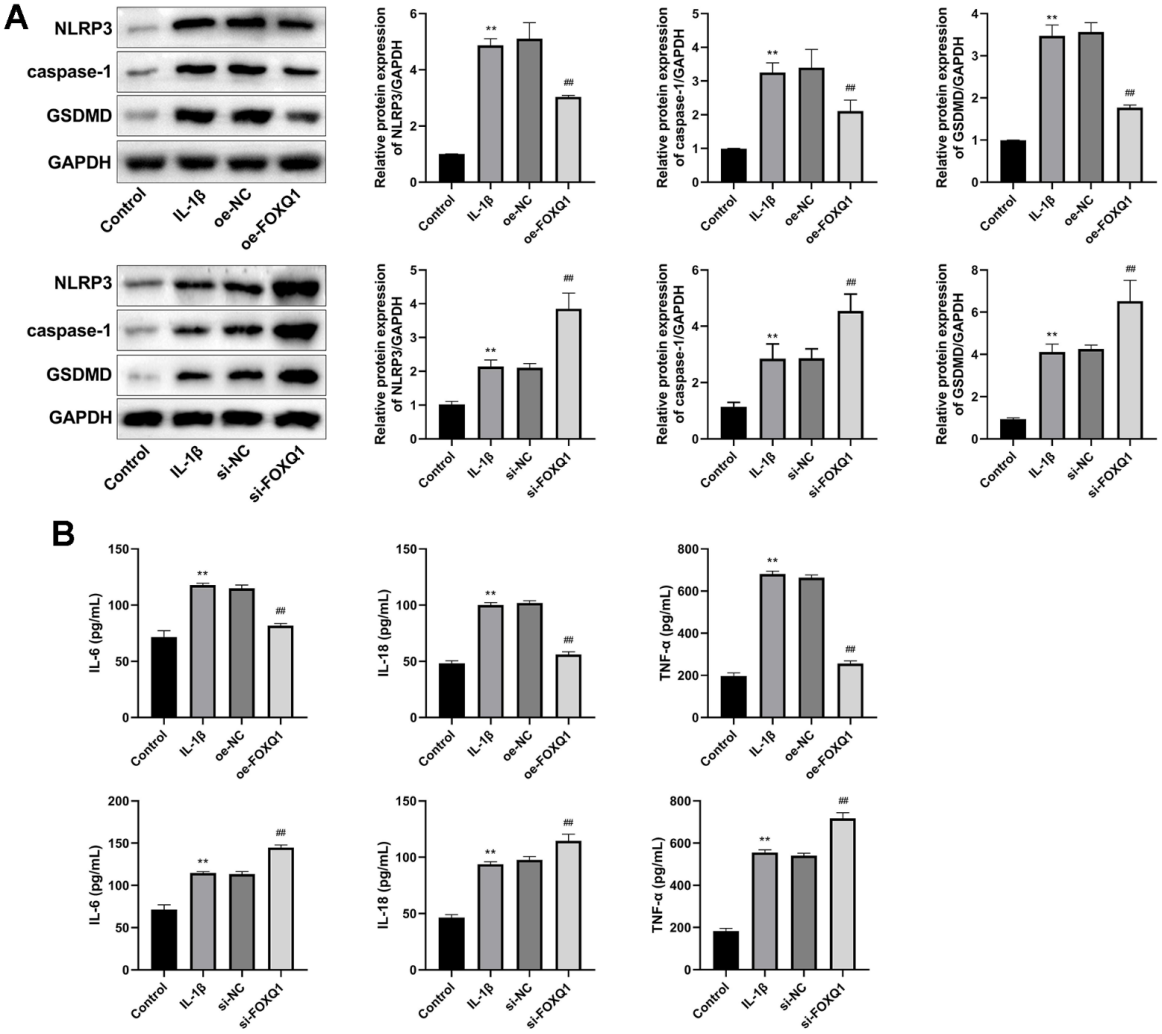
**FOXQ1 affects pyroptosis.** (**A**) Western blot result of NLRP3, caspase-1, and GSDMD. (**B**) ELISA results of IL-6, IL-18, and TNF-α in serum. (^**^P < 0.01 vs. Control; ^##^ *P* < 0.01 vs. IL-1β).

We next investigated the FOXQ1 potential role in pyroptosis of ATDC5 cell model treated with IL-1β. Western blot showed that the relative expressions of NLRP3, caspase-1, and GSDMD in IL-1β group were increased compared with control group (*P* < 0.01). Compared with the IL-1β group, the expression levels of NLRP3, caspase-1, GSDMD were downregulated in IL-1β+ oe-FOXQ1 group, and upregulated in the IL-1β+ si-FOXQ1 group (Figure 7A, *P* < 0.01). Additionally, ELISA results revealed that the concentrations of inflammatory factors (IL-6, IL-18, and TNF-α) in IL-1β group were higher than those in the control group (*P* < 0.01). Overexpression of FOXQ1 reduced IL-6, IL-18, TNF-α levels, and FOXQ1 knockdown elevated these inflammatory cytokine levels (Figure 7B, *P* < 0.01). All these findings indicated that FOXQ1 could inhibit pyroptosis in ATDC5 cells treated with IL-1β.

## DISCUSSION

This study explored the connection between FOXQ1 and OA in the OA model. We performed an NGS for articular cartilage to detect the transcriptome difference between the OA model and controls, and we validated and confirmed that the expression of FOXQ1 was inhibited in the articular cartilage of the OA model. In subsequent experiments in the ATDC5 cellular model, we proved that FOXQ1 increased cell viability and decreased apoptosis of IL-1β induced ATDC5 cells. Finally, the mechanism study suggested that the expressions of NLRP3, Caspase-1, and GSDMD were associated with FOXQ1 in IL-1β induced ATDC5 cell model. These findings suggested that FOXQ1 might be involved in OA and overexpression of FOXQ1 might postpone the progression of OA.

Eight candidate DEGs were confirmed by using qPCR, including four up-regulated genes (*Adrb3*, *Adig*, *Megf6*, *C1qtnf3*) and four down-regulated genes (*Isoc2b*, *Heatr4*, *Foxq1*, *Misp*). Adrb3, a member of the family of β adrenergic receptors, mediates the activation of adenylate cyclase caused by catecholamine via the action of G proteins and participates in the regulation of lipolysis and thermogenesis [26]. Adig is involved in the positive regulation of fat cell differentiation, and regulated chondrocyte adipogenic differentiation in OA [27]. Megf6 is located in a collagen-containing extracellular matrix and regulates zebrafish’s cartilage and bone formation [28]. C1qtnf3 relieves the inflammation induced by LPS through the PPAR-γ/TLR4 pathway [29] and improves the proliferation of chondrogenic precursors and chondrocytes during chondrogenic differentiation [30]. The hypomethylation of Isoc2b is associated with age in mice [31], but studies on its function are still lacking. Heatr4 is predicted to possess oxidoreductase activity and is orthologous to human HEATR4. Misp, an actin-bundling protein plays a part in controlling cell morphology and mitotic progression and regulates the immune infiltration of pancreatic ductal adenocarcinoma [32]. These genes are related to cartilage differentiation or immunity, and further mechanism study needs to be performed in the future.

Transcription factor FOXQ1 has been involved in epithelial-mesenchymal transition (EMT), cell cycle, cell proliferation, and regulation of senescence-associated inflammation [19]. We performed an IL-1β induced ATDC5 cell as an OA cell model to explore the role of FOXQ1 in OA. Caspase-1/11 induced pyroptosis is a kind of programmed cell death, which can be activated by pattern recognition receptors, and triggers the release of IL-1β and IL-18 [33]. There have been found that inhibiting pyroptosis of synovial macrophages can reduce synovial inflammation and fibrosis in OA mice model, indicating that pyroptosis participates in the progression of OA [34]. Knockdown of miR-155 inhibits chondrocyte pyroptosis by targeting SMAD2 in OA model [35]. MiR-140-5p inhibits pyroptosis of chondrocytes to alleviate OA cartilage damage by suppressing CTSB/NLRP3 [36]. In this study, we found that upregulation of FOXQ1 enhanced viability, weakened apoptosis of chondrocytes, and FOXQ1 knockdown exerted the opposite trend. Meanwhile, we detected that the expression of FOXQ1 was negatively relevant to pyroptosis-related proteins including NLRP3, Caspase-1, GSDMD, and inflammatory cytokines containing IL-6, IL-18, and TNF-α, denoting that overexpression of FOXQ1 alleviated the progression of OA by inhibiting pyroptosis.

Although the pathogenesis has not been revealed clearly, it has been a consensus that OA is caused by the imbalance between the repair and destruction of joint tissue [37]. Meanwhile, cartilage degeneration is a typical characteristic lesion of OA, so delaying degeneration and promoting regeneration are the two interventions currently [38]. So far, there are few studies on the role of FOXQ1 in osteogenesis. As a nucleic acid binding protein, FOXQ1 can inhibit the replicative senescence by decreasing the levels of IL-6 and IL-8 via modulation of the SIRT1-NFκB pathway [39], and the FOXQ1-ANXA2 complex can promote the Wnt/β-catenin pathway in bone MSCs, thereby leading the osteogenic differentiation subsequently [40]. In addition, Xia et al. have found that silencing of FOXQ1 significantly inhibits the osteogenic differentiation of bone-derived MSCs from osteoporosis with T2DM [25]. Based on the previous study results, FOXQ1 could be seen as a chondrocyte biogenesis factor in osteogenesis, but the role of FOXQ1 in cartilage degradation of OA is still unclear. NLRP3-induced pyroptosis of chondrocytes and synoviocytes have been proved as a common osteoclasia process in OA [12, 33]. Pyroptosis always promotes cartilage degradation, and it has been found that some NLRP3 inhibitors can protect against cartilage degradation in OA, including MCC950 [41], icariin [42], Nrf2 [43], and so on. So pyroptosis has been considered as an intervention target in the progression of OA, which always promotes cartilage degradation [12]. The inhibitory affection of FOXQ1 on NLRP3-induced pyroptosis may make it a new target for the therapy of OA. The mechanism of FOXQ1 regulating pyroptosis needs to be further investigated in animal and cellular models.

## CONCLUSIONS

In the present research, we found that FOXQ1 retarded OA progression via down-regulating pyroptosis induced by NLRP3. Further studies on the role of FOXQ1 in OA may help to develop effective targeted drugs, providing a novel option for the therapy of OA.

## Supplementary Material

Supplementary Tables
