# Supplementary material for: FOXQ1 inhibits the progression of osteoarthritis by regulating pyroptosis
Source: Aging (Albany NY). 2024 Mar 19;16(6):5077–90. doi: 10.18632/aging.205600 (PMC11006491; doi:10.18632/aging.205600)
Supplement: Supplementary Tables [file aging-16-205600-s001.pdf]

SUPPLEMENTARY TABLES

Supplementary Table 1. The sequences of primers and siRNAs for QRT-PCR validation.

Name	Sequence (5'-3')
GAPDH-F	GAGAGTGTTTCCTCGTCCCG
GAPDH-R	ACTGTGCCGTTGAATTTGCC
ADRB3-F	AAACGGCTCTCTGGCTTTGT
ADRB3-R	ATTACCAGCAGGTTGCCTCC
ADIG-F	GTGAGCGACCTCACACTCTC
ADIG -R	CACAGCCACACTCAGATGGT
MEGF6-F	GCATTGACATCGACGACTGC
MEGF6-R	AGCCGTCTGTGTTGAGTCTG
C1QTNF3-F	TGGCCAAAGGAGATGAAGTCT
C1QTNF3-R	GAAAGCCTGCGAAGGTGGAG
ISOC2B-F	TCCCAGAGTCCTCCATCCTG
ISOC2B -R	TGGACAGAAAGACGCCACTC
HEATR4-F	TAGGGTCCAGGCCATCATCA
HEATR4-R	CCGCTCCTGGGAATTTCCTT
FOXQ1-F	ACTGATGACAGCAGAACGCA
FOXQ1-R	AGGTGTATTCGCTGTTGGGG
MISP-F	TCGCTGAGCTCAAAGCAAGA
MISP-R	CTGGGACCTTGAATCGCAGT
Si-FOXQ1-1	CGCGGACTTTGCACTTTGA
Si-FOXQ1-2	AGGGAACCTTTCCACACTA
Si-FOXQ1-3	CCATCAAACGTGCCTTAAA
Si-NC	TTCTCCGAACGTGTCACGT

Supplementary Table 2. Top 15 up and down-regulated differential expressed genes.

Genes	Description	log2fc	pval	padj	Up/down
Adrb3	adrenergic receptor, beta 3	5.234	0.006	0.154	Up
Xlr3b	X-linked lymphocyte-regulated 3B	4.359	0.000	0.034	Up
Mrgprb2	MAS-related GPR, member B2	3.911	0.035	0.394	Up
1700001O22Rik	RIKEN cDNA 1700001O22 gene	3.902	0.049	0.462	Up
Slc7a10	solute carrier family 7	3.666	0.005	0.139	Up
Tmem139	transmembrane protein 139	3.444	0.021	0.310	Up
Serpinc1	serine (or cysteine) peptidase inhibitor, clade C (antithrombin), member 1	3.343	0.026	0.342	Up
Moxd1	monooxygenase, DBH-like 1	3.317	0.007	0.182	Up
Adig	adipogenin	3.158	0.004	0.130	Up
Pou2f3	POU domain, class 2, transcription factor 3	3.129	0.044	0.441	Up
Cfd	complement factor D (adipsin)	3.098	0.003	0.110	Up
Megf6	multiple EGF-like-domains 6	2.854	0.026	0.344	Up
C1qtnf3	C1q and tumor necrosis factor related protein 3	2.819	0.006	0.161	Up
Lgals12	lectin, galactose binding, soluble 12	2.789	0.038	0.409	Up
Cidec	cell death-inducing DFFA-like effector c	2.784	0.012	0.228	Up
2010109A12Rik	RIKEN cDNA 2010109A12 gene	-3.042	0.019	0.294	Down
Isoc2b	isochorismatase domain containing 2b	-2.754	0.000	0.026	Down
Tdrd5	tudor domain containing 5	-2.537	0.005	0.147	Down
Heatr4	HEAT repeat containing 4	-2.461	0.050	0.465	Down
Foxq1	forkhead box Q1	-2.225	0.027	0.351	Down
Gvin1	GTPase, very large interferon inducible 1	-2.197	0.005	0.145	Down
Misp	mitotic spindle positioning	-2.138	0.026	0.347	Down
Gm8909	predicted gene 8909	-2.136	0.026	0.347	Down
Gipr	gastric inhibitory polypeptide receptor	-2.064	0.045	0.448	Down
Gm4841	predicted gene 4841	-2.045	0.032	0.380	Down
Slc4a5	solute carrier family 4, sodium bicarbonate cotransporter, member 5	-2.038	0.040	0.420	Down
Olfr433	olfactory receptor 433	-1.934	0.012	0.230	Down
Cdcp3	CUB domain containing protein 3	-1.903	0.027	0.353	Down
H2-Q7	histocompatibility 2, Q region locus 7	-1.888	0.002	0.095	Down
Kcnip2	Kv channel-interacting protein 2	-1.873	0.045	0.448	Down
